# Hybridization thermodynamics of NimbleGen Microarrays

**DOI:** 10.1186/1471-2105-11-35

**Published:** 2010-01-19

**Authors:** Ulrike Mueckstein, Germán G Leparc, Alexandra Posekany, Ivo Hofacker, David P Kreil

**Affiliations:** 1 WWTF Chair of Bioinformatics, Boku University Vienna, Muthgasse 18, 1190 Vienna, Austria; 2 Theoretical Biochemistry Group, Institute for Theoretical Chemistry, University of Vienna, Währingerstrasse 17, 1090 Vienna, Austria

## Abstract

**Background:**

While microarrays are the predominant method for gene expression profiling, probe signal variation is still an area of active research. Probe signal is sequence dependent and affected by probe-target binding strength and the competing formation of probe-probe dimers and secondary structures in probes and targets.

**Results:**

We demonstrate the benefits of an improved model for microarray hybridization and assess the relative contributions of the probe-target binding strength and the different competing structures. Remarkably, specific and unspecific hybridization were apparently driven by different energetic contributions: For unspecific hybridization, the melting temperature *T_m _*was the best predictor of signal variation. For specific hybridization, however, the effective interaction energy that fully considered competing structures was twice as powerful a predictor of probe signal variation. We show that this was largely due to the effects of secondary structures in the probe and target molecules. The predictive power of the strength of these intramolecular structures was already comparable to that of the melting temperature or the free energy of the probe-target duplex.

**Conclusions:**

This analysis illustrates the importance of considering both the effects of probe-target binding strength and the different competing structures. For specific hybridization, the secondary structures of probe and target molecules turn out to be at least as important as the probe-target binding strength for an understanding of the observed microarray signal intensities. Besides their relevance for the design of new arrays, our results demonstrate the value of improving thermodynamic models for the read-out and interpretation of microarray signals.

## Background

Microarrays have become the predominant method for studying gene expression on a genomic scale. It has been recognised, however, that probes interrogating different regions of the same mRNA target show considerable variation in signal intensities [1,2], and that the observed intensity variation is highly sequence-dependent [3-5]. This is expected because different probes vary in their tendency of forming intraand intermolecular structures that compete with the hybridization of the probe-target duplex, resulting in different hybridization efficiencies [6,7]. Comparative studies have indicated that accurate thermodynamic models based on the physico-chemical parameters underlying probe-target interactions are particularly good predictors of actual probe binding behaviour and thus microarray signal intensity [8]. This is not only important in the design of new arrays, where specific and uniform probes need to be selected [9], but also for the readout of data from established platforms, where a better comparability of signals from different genes improves quantitative modelling and the sensitive detection of subtle higher-dimensional patterns. When the effective hybridization temperature is not known, the probe-target melting temperature *T_m _*is often calculated to predict the expected thermodynamic stability of the hybridized complex [10,11]. The melting temperature is still one of the most popular measures in the evaluation of microarray probes. It gives the temperature at which half of all probes form a duplex with their target while the other half are unbound, assuming a simple two state transition, thus providing information about the probe binding behaviour at the *melting *temperature. It makes, however, no statement about the binding affinity at the actual hybridization temperature. Probe-target dimers with the same *T_m _*can actually behave quite differently at typical reaction temperatures, which are usually considerably lower than the *T_m _*[12-14]. Increasingly, general thermodynamic models of probe-target hybridization have become established in the prediction of microarray probe behaviour. These models are either based on published thermodynamic rules for nearest-neighbour base-pairing and experimentally determined parameters, or they determine model parameters from fits of the observed probe intensities [1-4]. The free energy of probe-target hybridization can so be calculated for the effective hybridization temperature.

None of the above approaches yet considers that nucleic acid sequences can form stable intramolecular structures that compete with the formation of the probe-target duplex: Only freely accessible, *i. e*., monomeric and unfolded probe and target molecules can interact to form probe-target dimers [13,15-17]. More elaborate models therefore include the effects of the secondary structures of the probe [8,18-20] and the target [9,21,22] on the overall binding efficiency. Another factor that reduces the efficiency of probe-target hybridization is the formation of probe-probe dimers [6].

Wei *et al*. [23] recently examined to what degree several probe properties affect microarray signal intensities. In this work we extend this analysis by additionally determining the influence of target secondary structure, the free energy of probe-target hybridization, and the strength of probe-probe dimerization on the overall efficiency of probe-target binding on microarrays. We used a partition function approach to capture the full dynamic potential of the different inter- and intramolecular interactions [24,25]. Although the stability of the probe-target duplex alone is a good first indicator of hybridization behaviour, we will show that the signal variation observed in the examined data sets can be better explained by the *effective interaction energy*. The effective interaction energy, which is the free energy of probe-target hybridization reduced by the free energies of probe and target secondary structures and the probe-probe duplex formation, can predict probe dependent signal intensity variation twice as well as the melting temperature *T*_*m*_. Considering a complementary tiling array study [26], we can moreover show that the higher predictive power of the effective interaction energy is independent of the typical probe length and the type of target nucleic acid (cDNA or RNA) in an experiment.

## Methods

### Microarray data

We studied the sequence-dependent intensity variations for two different tiling array experiments. The first one features sets of probes targeting different regions of the same transcripts (Dataset II of Wei *et al*. [23]). It comprises nine tiling arrays with a resolution of 22 nt, each containing about 385,000 probes interrogating the expression of 32,424 regions throughout the genome. Probe length ranged from 45 to 75 bases. Chips had been manufactured with 5 nt thymidine linkers and had been hybridized to cDNAs from undifferentiated human Embryonic Stem Cells (hESCs) by NimbleGen Systems [27]. Raw expression data had been extracted using NimbleScan software v2.1. After qspline normalization, a non-linear method for controlling signal-dependent sources of variability [28], data were median centred using control set probe intensities. For comparability to the original study, our analysis is based on the same preprocessed data. Probe targets were identified by WU-BLAST (W.Gish, pers. comm.) run against UCSC 'Known Genes' annotation [29] (as obtained 2008-08-29). To efficiently identify perfect matches, WU-BLAST parameters were set as follows: seed alignment word size W = probe length, match score M = 1, mismatch score N = -1, gap penalty Q = 3 and gap extension penalty R = 1. As expected for a tiling array, many probes had no cDNA target (about 90%), and about half the matches were on the reverse strand. For simplicity and to avoid confounding effects, we have focussed on probes complementing the sense strand and without matches to multiple genes. For simplicity, we only included probes that showed no cross-hybridization to any mRNA in the UCSC 'Known Genes' annotation [29] in our analysis.

The second experiment used 25-mer oligonucleotides for perfectly matching 1 nt tiling probes of ribosomal RNA (rRNA) sequences from nine nematodes [26]. The rRNA targets were generated by *in vitro *transcription. Each rRNA target was separately hybridized to the specific compartment on the 12-well NimbleGen array, *i. e*., no interference between targets was possible. We analyse the fluorescence intensities of the probes perfectly matching the single hybridized target, giving purely specific signals with no cross-hybridization. In the hybridization reactions, an rRNA concentration of 375 ng was used for each target [26].

### Calculation of thermodynamic parameters and model choice

#### Melting temperature

Following the approach of Wei *et al*. [23], melting temperatures were computed by the SantaLucia *et al*. [15] method, and included a helix-initiation factor and corrections for sodium ion and Formamide concentrations [30]:

where [*Na*^+^] = 0.6 M is the sodium ion concentration in molar units, *A *is a helix initiation factor equal to *-*10.8 cal/(KM), *C*_*t *_the molecular concentration of the oligonucleotide strands in molar units, *F *the correction term for Formamide, namely 0.63°C *per *1% Formamide. The universal gas constant *R *= 1.987 cal/(KM). Wei *et al*. [23] estimated the oligonucleotide concentration to *C*_*t *_= 6.1 * 10-^17^*M *and used a Formamide concentration of 35% for hybridization (Ron Stewart, pers. comm., 2009). Since we were interested in the sensitivity of our study to *T_m _*calculation variations, we also computed the *T_m _*for different settings of molecular concentration *C*_*t*_, and with or without Formamide and sodium correction terms.

#### Probe secondary structure

To facilitate a comparison of results with the original study [23], the minimal free energy (mfe) of the secondary structure of the probe molecule was computed by hybrid-ss-min [31,32], with the nucleic acid type (option -NA) set to 'DNA'.

#### Multi-state thermodynamic model

Many established thermodynamic models use the free energy of the most stable predicted secondary structure, *i. e*., the minimal free energy (mfe) structure [31,33,34]. The hybridization of nucleic acid molecules, however, is dynamic and each molecule exists in an ensemble of structures [24,35]. An accurate prediction of nucleotide behaviour can therefore be achieved with a partition function approach [36] established in the field of statistical mechanics. For thermodynamic prediction of probe-target interaction we thus used *RNAup *[25]. *RNAup *calculates free energies applying the full partition function over possible inter- and intramolecular structures of a probe and its target. *RNAup *calculations employed the energy parameters for DNA folding as distributed with D. Mathews' RNAstructure [33] in the calculation of thermodynamic models for the data set of Wei *et al*. [23]. For the data set of Pozhitkov *et al*. [26] we used the default RNA folding parameters in the calculation of secondary structures of the rRNA target and the DNA folding parameters as distributed with D. Mathews' RNAstructure in the calculation of secondary structures of the DNA probe and for the assessment of probe-probe dimers. In calculating the probe-target binding energy we used the RNA-DNA folding parameters from Sugimoto *et al*. [37,38]. *RNAup *models the interaction between a probe and its target as a stepwise process. The first step of the calculation computes the free energies Δ*G*_*p *_and Δ*G*_*t *_that are needed to unfold the secondary structures of the probe and its binding site in the target, respectively. Then the binding energy Δ*G*_*h *_gained by hybridization of the probe to its target is calculated. The total binding energy is then given by Δ*G*_*h*_-Δ*G*_*p*_-Δ*G*_*t*_. In addition we consider the free energy Δ*G*_*pp*_, that is necessary to unfold probe-probe dimers within the same probe feature, and compute it in an independent step using *RNAup*. The effective interaction energy Δ*G *is finally given by subtracting the free energies needed to open secondary structures and unfold probe dimers from the binding free energy:(1)

Here Δ*G*_*h*_, Δ*G*_*p*_, Δ*G*_*t*_, and Δ*G*_*pp *_are always ≤ 0. For unstable structures, their respective contributions were zero. The final effective interaction energy Δ*G *was stable (< 0) for all considered probe-target pairs.

#### Target secondary structure

The probe binding site area in the target may form structural motifs with bases some distance from the probe binding site. To calculate the free energy Δ*G*_*t *_of unfolding structures in the target we therefore consider a target fragment including the probe binding site plus parts of the flanking sequence. Koehler *et al*. [21] showed that 90% of base pairs are formed between nucleotides less than 85 bases apart in the primary sequence. They suggest that over 90% of the predicted structures of the full length target can be found by using a target fragment consisting of the probe binding site flanked by 170 bases on either side. We can thus safely use a target fragment including flanking regions of 200 bases on either side of the probe binding region for the calculation of the free energy Δ*G*_*t *_that is required to unfold the probe binding site in the target.

### Importance ranking of thermodynamic parameters

Wei *et al*. [23] used the GUIDE algorithm [39] to rank the thermodynamic parameters in order of their importance for predicting signal intensity. GUIDE constructs a non-linear model fit by finding an optimal partitioning of the data together with piecewise least-square regression models on each data subset, forming a so-called regression-tree. Variables for the split-condition of a tree-node are selected by unbiased detection of pairwise interactions and curvature, where the split points are found by exhaustive search. Over-partitioning/over-fitting of the data is avoided by limiting the size of the final regression tree by cost-complexity pruning in cross-validation, selecting the smallest tree with a mean prediction error within one standard error of the minimal prediction error achieved. The algorithm also provides importance scores, which reflect the contribution a predictor variable makes to the non-linear model. For each split-node and variable in the regression-tree, it is computed from the chi-square statistic of the interaction/curvature detection step for the underlying piecewise-constant regression model, and then weighted by the square-root of the data subset size at the split-node. The overall importance score of a predictor is obtained by summing over all split-nodes of the tree (Wei-Yin Loh, pers. comm., 2009). Software and further bibliography are available from http://www.stat.wisc.edu/~loh/guide.html. For direct comparability to earlier work, we also employ GUIDE in this study. Bootstrapping was used to study the robustness of relative importance and obtain error estimates for GUIDE results: For the whole dataset of about 3 million probes, 100 random subsets of 200,000 probes each were sampled with replacement from the original data. For the smaller subsets examined (*cf*. Results), 100 random subsets were generated, each containing 90% of all probes in the respective dataset. For the GUIDE scores separately obtained from each of the 100 random subsets, means and standard deviations were computed, which were finally scaled by the mean for the most important parameter to give the relative importance scores. The analysis was repeated both on the raw fluorescence intensities and on the log-intensities (Additional File 1).

## Results

### 0.1 Thermodynamics of microarray hybridization

We used tiling microarray data from Wei *et al*. [23] to study the influence of inter- and intramolecular structures on the efficiency of probe-target duplex formation. For this purpose, tiling arrays have the advantage that probes have been selected only by the tile start position but no probe selection to optimize hybridization efficiency or uniformity has been applied [5]. One therefore has a set of probes with a highly varying potential for intra- and intermolecular structure formation, which results in different binding efficiencies and thus microarray hybridization signals for the same transcript target, allowing a systematic investigation of probe specific effects.

Our analysis followed the methodology of Wei *et al*. [23] yet newly introduces target-side analysis. In addition, we consider an accurate representation of the free energy of probe-target dimerization and introduce an extended model for microarray hybridization that includes probe dimerization. Our approach assesses the influence of inter- and intramolecular structures that compete with probe-target duplex formation in the hybridization process. We subsequently analyse the relative contributions of the competing structures and the probe-target duplex binding strength on the measured signal intensities.

#### Thermodynamics of non-specific hybridization

**Figure 1:**
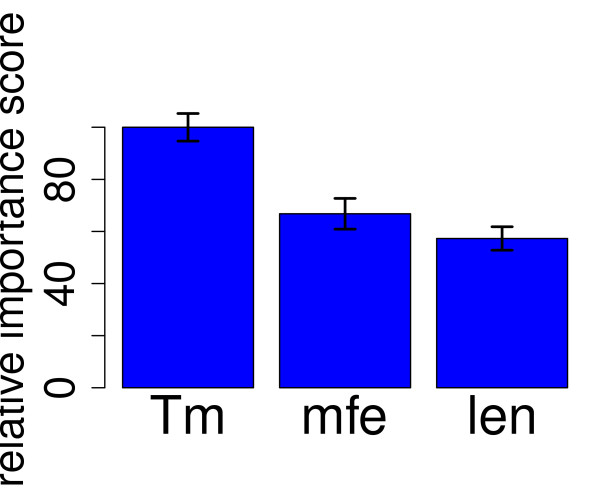
**Importance ranking of *T_m_*, the minimal free energy (mfe) of probe structure, and probe length**. All probes: Importance ranking of *T_m_*, the minimal free energy (mfe) of probe structure, and probe length. Here, 'Tm' stands for the melting temperature, 'mfe' labels the minimal free energy of probe secondary structure and 'len' the probe length. Probe lengths ranged from 45 to 74 nt. Error bars are from 100 random bootstrap samples of 200,000 probes each.

As a first step we aimed to reproduce the results of the original study and thus calculated the melting temperature *T_m _*and the minimal free energy (mfe) of probe secondary structure for each probe. We determined the relative importances of *T*_*m*_, the mfe of probe structure, and the probe length for the prediction of probe signal intensities according to the GUIDE algorithm [39], validated by bootstrap. The importance ranking for *T*_*m*_, mfe, and probe length (Fig. 1) were in agreement with the original results of Wei *et al*. [23]: For the whole dataset, *T_m _*was the best predictor of intensity variation, followed by the mfe of probe secondary structure. Probe length was the least relevant parameter.

The probes in this dataset are tiling probes designed to interrogate the entire human genome. In a hybridization to cDNAs, the majority (about 90%) of these tiling probes do not have a transcript target. These probes reflect non-specific hybridization and they dominate the dataset. The obtained importance ranking actually did not change when only considering probes with non-specific hybridization (Additional File 2: Fig. A.2). We therefore observe that the melting temperature *T_m _*is the best predictor of non-specific hybridization signal. (For these probes with no known transcript target, more advanced thermodynamic models like the effective interaction energy cannot be calculated, as they require knowledge of the target sequence.)

**Figure 2:**
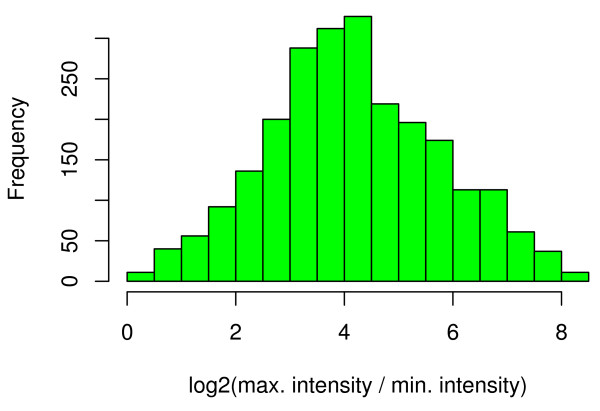
**Probe specific intensity variation**. Probe specific intensity variation. For each target, the probe specific intensity variation is assayed as log_2_(max. intensity/min. intensity). The histogram shows the distribution observed for 2,472 clearly expressed target genes.

#### Thermodynamics of specific hybridization

Consequently, it is interesting to focus on the subset of probes that target expressed genes for a complementary examination of predominantly specific hybridization signals. For this purpose, probes were selected that matched the plus strand of single genes from the UCSC 'Known Genes' annotation [29]. For the identification of clearly expressed genes, we employed a conservative strategy of keeping only targets having at least one probe with a signal higher than the mean intensity of probes targeting transcripts. (Results, however, were robust under different threshold choices for target expression; see Additional File 3, Fig. A.3.)

**Figure 3:**
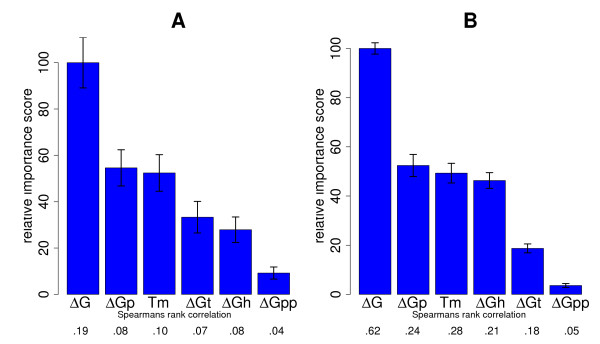
**Importance ranking of thermodynamic probe properties including target-side modeling**. Importance ranking of thermodynamic probe properties including target-side modelling. The left hand figure (A) shows the Guide ranking for the clearly expressed genes showing no cross-hybridization from Wei *et al*. [23], whereas the right one shows the results for the Pozhitkov *et al*. [26] dataset (B). ΔG stands for the effective interaction energy including relevant competing intra- and inter-molecular effects. ΔG_p _labels the free energy of the probe secondary structure, ΔG_t _the free energy of the probe binding site secondary structure in the target, ΔGh the free energy of the probe-target duplex, and ΔGpp the free energy of probe-probe dimerization within the same probe feature. 'Tm' stands for the melting temperature. Error bars are from bootstrap re-sampling of 90% of all probes. Below the *x*-axis, the Spearman rank correlation of predictions to the observed signal intensity is shown. All correlations were highly significant, with the correlation for ΔGpp in (A) having *p *< 10^-9^, and *p *≤ 10^-5 ^in (B). For the other correlations, *p *< 10^-15^. We note that the correlation values for the sample with complex background are considerably lower, suggesting further scope for improvements in our model.

By these criteria, we found 2,472 clearly expressed transcripts, interrogated by 74,267 probes, with an average of 30 probes per target. The intensity of probes targeting the same transcript can vary up to 300-fold, as shown in Fig. 2: Probe specific intensity variation was assayed as the log_2_-ratio of the maximal and the minimal probe intensities for each transcript. Averaged over all 2,472 transcripts, we observed a typical 18-fold max/min probe intensity variation (mean log-ratio of 4.2).

Considering probes for known targets now also allows an introduction of target-side modelling. For simplicity, we further focus on probes with no cross-hybridization potential (*cf*. Additional File 4). In our extended model we relate the observed binding efficiency to an effective interaction energy Δ*G*. This is obtained by subtracting the free energies of inter- and intramolecular interactions that interfere with the formation of probe-target dimers from the free energy gained by probe-target dimer hybridization Δ*G*_*h*_:(2)

where Δ*G*_*p *_is the free energy of the probe secondary structure, Δ*G*_*t *_the free energy of the probe binding site secondary structure in the target, and Δ*G*_*pp *_the free energy of probe-probe dimerization within the same probe feature. For most probes and targets considered, stable alternative structures were observed (Additional File 5, Table A.1), highlighting the importance of considering the effects of this competition for the binding of the probe.

The importance of alternative thermodynamic properties in a prediction of probe signal intensity according to the GUIDE algorithm was validated by bootstrap as before. Fig. 3 shows that the effective interaction energy Δ*G *is by far the best predictor of signal intensity for specific hybridization, with about twice the relative importance score compared to alternative predictors like the melting temperature *T_m _*(left panel). In order to test the generic nature of this result, we also examined a complementary tiling array experiment [26] that probes several ribosomal RNAs (rRNAs) with 25 nt oligonucleotides. Besides providing a test case with much shorter probes and a different target type (RNA instead of DNA), hybridization conditions were simpler in this experiment, featuring uniform target concentrations and no complex background (and thus no cross-hybridization). Table 1 compares the two data sets. Also in this very different experiment, the effective interaction energy Δ*G *was the best predictor of signal intensity, clearly outperforming alternative predictors like *T_m _*(Fig. 3, right panel).

**Table 1:** Characteristic differences between the two studied datasets

Comparison of datasets		
**Parameter**	**Pozhitkov *et al***. [26]	**Wei *et al***. [23]

probe	DNA	DNA
target	rRNA	cDNA
probe length	25-mers	45- to 75-mers
resolution	1 nt	22 nt
number of probes	7, 519^a^	21, 813^b^
number of targets	9^a^	2, 472^b^
target concentration	375 ng	Unknown

^a ^We analysed the probes perfectly matching the respective separately hybridized individual targets, ensuring no crosshybridization effects.

^b ^We focused on probes targeting clearly expressed transcripts and showing no cross-hybridization in our analysis.

Caption: This table summarizes the characteristic differences between the two datasets studied. The dataset of Pozhitkov *et al*. [26] comprises tiling probes targeting 9 rRNA fragments. The dataset of Wei *et al*. [23] consists of tiling probes designed for high-resolution interrogation of the entire human genome. The row 'probes' shows the nucleic acid type of probes, row 'target' the nucleic acid type of targets.

**Figure 4:**
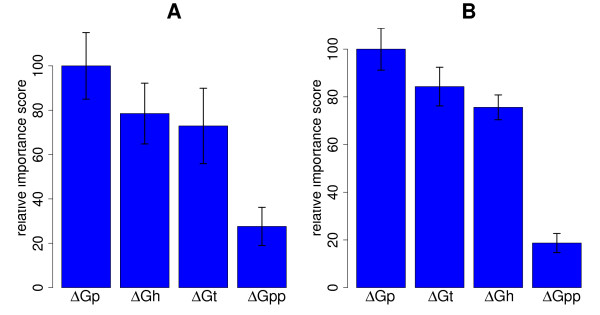
**Importance ranking of contributions to Δ*G***. Importance ranking of Δ*G*_*h*_, Δ*G*_*p*_, Δ*G*_*t*_, and Δ*G*_*pp *_computed separately. The left-hand side (**A**) shows the GUIDE results for the data from Wei *et al*. [23] (probes for known targets without cross-hybridization), the right-hand side the results for Pozhitkov *et al*. [26] dataset (**B**).

The relative importance of the other predictors is most cleanly examined in a separate ranking run. The rank orders obtained for the two data sets was again very similar. Although the order of Δ*G*_*h *_and Δ*G*_*t *_was switched, their relative important scores were separated by less than one standard deviation. We could thus conclude that Δ*G*_*p*_, Δ*G*_*h*_, and Δ*G*_*t *_were all of similar importance, whereas Δ*G*_*pp *_was considerably less influential (Fig. 4). Even though Δ*G*_*pp*_, the free energy of probe-probe dimerization, was by itself the least descriptive parameter, its inclusion in Δ*G *improved results by a further 10% (Additional File 6: Fig. A.5). The similar relevance of competing intramolecular structures can be understood by considering the extreme observed variabilities of their contributions for probes of comparable Δ*G*. Table 2 shows the variation of the free energies of these structures for probes with typical effective interaction energies Δ*G *± 1 kcal/mol. Even for probes of very uniform Δ*G*, which was shown to be a good predictor of probe signals, the variations observed for Δ*G*_*h*_, Δ*G*_*p*_, and Δ*G*_*t *_nearly match the ranges seen across all the probes for clearly expressed genes.

**Table 2:** Variation of thermodynamic properties for probes of similar effective interaction energies or binding strengths.

Variation of properties for probes with similar free energies				
	**Variation range within ± 1 unit of the median**			
	**fixed Δ*G***	**fixed Δ*G***_*h*_	**fixed *T***_*m*_	**all probes**

-Δ*G*	**44 **... **46**	26 ... 62	22 ... 66	19 ... 66
-Δ*G_p_*	0.5 ... 18	0.1 ... 16	0.3 ... 16	0.1 ... 22
-Δ*G_t_*	2.2 ... 23	2.3 .... 26	1.7 ... 22	0.6 ... 32
*T_m_*	36 ... 64	40 ... 60	**46 **.... **48**	33 ... 66
-Δ*G_h_*	54 ... 84	**65 **... **67**	49 ... 89	45 ... 89

Caption: Variation of thermodynamic properties for probes of similar effective interaction energies or similar probe-target duplex binding strengths. Probes similar in certain thermodynamic properties were selected from an interval of ± 1 of the median values of Δ*G *(column 1), Δ*G*_*h *_(column 2), and *T_m _*(column 3). Intervals are printed in bold face. These intervals contained 13460, 9806 and 10494 probe-target pairs, respectively. The last column, labelled 'all probes', shows the variation range for all probes matching clearly expressed genes. Less than 2% of all probes had unstable probe-probe interactions.

In summary, the consideration of multiple inter- and intramolecular structures that compete with the formation of a probe-target duplex improves the prediction of probe signal intensity significantly, reflecting particularly the importance of probe and target accessibility for microarray hybridization.

### Influence of steric effects and probe synthesis yield

Microarray probes are attached to the chip surface with one end, the other end protrudes into solution. This causes different reaction conditions for the two ends of a probe. In high-density microarrays the surface-attached end is less accessible than the end in solution due to steric restrictions caused by the substrate [40] and crowding effects due to neighbouring probes [41]. Therefore the end of the probe that protrudes into solution plays a larger role in hybridization than the surface-tethered end [23,42].

**Figure 5:**
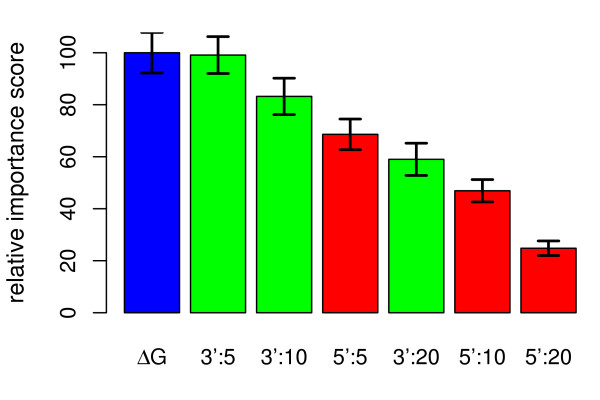
**Effect of removal of terminal bases from the probe sequence**. Effect of removal of terminal bases from the probe sequence. Δ*G *is the effective interaction energy of the full-length probe. For shortened probes, the respective end (5' is the solution end, 3' is the surface-tethered end) and the number of bases removed (5, 10, 20) are given, *i. e*., 3': 5 refers to the removal of 5 bases from the tethered (3') terminus.

To study these effects using our extended model, we removed the terminal 5, 10, or 20 nucleotides from both ends of the probe sequence and reran the analysis with the shortened probes. Fig. 5 shows the comparison of the effective interaction energies of the shortened probes to Δ*G *of the full-length probes. While a removal of 20 and 10 bases at the surface end reduces the predictive power of Δ*G*, the effective interaction energy of probes shortened by 5 nt at the surface tethered end is as good as Δ*G *of the full length probes. This suggests that the 5 nt closest to the surface have only a marginal contribution to the effective interaction energy.

The influence of the terminal bases at the solution end can be limited by the synthesis yield. NimbleGen's mask-less array synthesis technology has an average stepwise yield between 96% and 98% [27]. The probes in this study had a length range of 45 *- *75 nt. Even for a coupling efficiency of 98%, less than 30% of 60-mer probes reach full length [12]. Nevertheless, we find that the effective interaction energies of probes shortened at the solution end were significantly less predictive than the values for full-length probes, even if only 5 nt were removed (Fig. 5). These results are in agreement with the observations of Wei *et al*. [23] that protruding ends contribute more to signal intensity than tethered ends, confirming the dominance of steric effects over limitations from synthesis yield for this platform.

## Discussion

Although DNA microarrays have become the predominant method for gene expression profiling, the quantitative understanding of the measurement process still constitutes an active field of research. It is recognised that probe signal variation is highly probe-sequence dependent [3-5]. A recent study [23] has thoroughly examined the effect of different probe properties on probe signal intensities. We have extended this work by introducing target side modelling, an accurate representation of the free energy of probe-target dimerization and an improved model for microarray hybridization that includes probe dimerization. We separately considered specific and unspecific hybridization modes.

Reproducing results of Wei *et al*. [23], we obtained the melting temperature *T_m _*as the best predictor of probe signal intensity for the employed tiling array (Fig. 1). Most tiling probes hit non-exonic regions, with only 10% of the probes targeting mature mRNAs. Most probes therefore showed non-specific hybridization, and these dominated the dataset. Indeed, working with all array probes or restricting analysis to the non-specific probes gave the same results (Additional File 2: Fig. A.2). We thus observe that *T_m _*is the best predictor of non-specific probe signals.

In contrast, focussing on clearly expressed genes, we could show that the effective interaction energy Δ*G *was the best predictor for specific probe signal intensity (Fig. 3). Here, we computed Δ*G *by also considering inter- and intramolecular structures that interfere with probe-target binding, *cf*. Eq. (1). This improved the prediction of signal intensity considerably, with Δ*G *performing about twice as well as *T*_*m*_. Probe and target secondary structure gave a similar performance for signal intensity prediction as the free energy of the probe-target duplex Δ*G*_*h *_or the melting temperature *T_m _*(Figs 3 and 4). The large impact of probe secondary structure can be understood by considering how secondary structure affects probe-target binding efficiency: Hybridization between two nucleic acids starts with the nucleation of a few perfectly matched bases, followed by a comparatively fast zipping reaction [43]. Nucleation can actually be the rate limiting step for hybridization [44]. Probe secondary structure reduces the number of available nucleation sites because bound bases cannot take part in a nucleation reaction [41]. Moreover, probes with secondary structure fold back on themselves, with the solution ends of the probes brought into closer proximity to the microarray surface. Steric effects close to the array surface are another considerable factor determining probe accessibility. All these effects reduce hybridization efficiency and thus probe signal and may explain the importance of probe secondary structure for probe signal intensity prediction. The large variation of Δ*G*_*t *_and its strong influence on probe signal, on the other hand, can be understood by considering that bases outside the binding site can also contribute to structures interfering with probe binding, which results in an overall larger number of potentially stable relevant secondary structures in the target. Finally, while the incorporation of probe and target secondary structures into an effective interaction energy, Eq. (1), made the biggest contributions to better model performance, the consideration of probe-probe interactions also improved prediction power by a further 10%.

Besides their relevance for the design of new arrays, our results have demonstrated the value of improving thermodynamic models for the read-out and interpretation of microarray signals. Necessary next steps in the development of improved models include both the incorporation of intermolecular target-target interactions as well as of 'cross-hybridization' effects to unintentionally matching non-target molecules in a complex sample background. Target-target interactions on one hand are particularly challenging because they involve multimers of a probe, its bound target, and another sequence that binds to the target. This other sequence can thus contribute to the measured signal intensity [45]. More accurate models of interactions with target sequences will also have to consider the respective target fragmentation and labelling steps of microarray protocols. Models of cross-hybridization on the other hand need to address two tasks: identifying potential non-targets unintentionally matching the probe, and modelling their influence on the hybridization signal. Established probe design tools already filter out non-specific probes through cross-hybridization prediction after efficient sequence-similarity based detection screens [46,47]; similar to the filtering employed in this study. Latest advances now promise sufficiently fast and more sensitive detection tools based on thermodynamic models [48,49]. While recent developments have shown how the competitive formation of probe and target secondary structures as well as probe-probe and target-target dimers affect the probability of finding the desired probe-target duplex [31], these calculations still require several hours of computation time *per *probe-target pair, precluding their large-scale application. In approximation, the free energy contributions of competing structures can be obtained separately and effective interaction energies can be calculated [25], as in Eq. (1). A similar approach could also be taken to quantitatively consider cross-hybridization effects, as implemented in state-of-the-art probe design tools [9]. For this, special validation experiments that allow a separation of specific signals and cross-hybridization effects will be valuable. The results of our current study suggest that the establishment and validation of more sophisticated models can in the near future provide further improvements to our understanding and ability to quantitatively predict microarray hybridization signals in a complex sample background.

## Conclusions

We have introduced an improved thermodynamic model for probe-specific signal intensity on microarrays. The hybridization efficiency between a probe and its targets is determined by the balance of the binding strength of the probe-target duplex on one hand and the competing formation of probe-probe dimers and secondary structures in either probes or targets on the other hand. Consequently, the effective interaction energy between a probe and its target is modelled as the free energy gained by probe-target duplex formation reduced by the free energies needed to open alternative structures competing with probe-target binding. For specific hybridization, the effective interaction energy is a twice as powerful predictor of signal intensity variation as the melting temperature *T*_*m*_. We furthermore analysed the effects of the alternative competing structures in relation to probe-target binding strength, which highlighted the strong influence of intramolecular structures on specific hybridization signals. In summary, the improved model introduced here considerably enhances our ability to predict and understand sequence-specific variation of microarray signal intensities.

## Authors contributions

UM performed the thermodynamic calculations and statistical analyses, and wrote the manuscript draft. GGL performed the sequence analyses for the identification of probe targets. AP supported UM in the statistical analyses. IH supervised the thermodynamic modelling work. UM and DPK were jointly responsible for the conception and design of the study. DPK supervised the statistical analysis. All authors closely collaborated in writing the final manuscript.

## Supplementary Material

Additional file 1 **Robustness of conclusions under scale transformation**. Fig. A.1 shows the importance ranking for thermodynamic properties of probes without cross-hybridization against known targets on different intensity scales (linear and logarithmic). Click here for file

Additional file 2 **Dominance of non-specific probes on the chip**. Fig. A.2 shows the importance ranking of Tm, the mfe of probe structure, and probe length for all probes that had no target in a mature mRNA. Click here for file

Additional file 3 **Effect of intensity threshold**. Fig. A.3 shows the importance ranking for thermodynamic properties of probes without cross-hybridization against known targets with different signal intensity thresholds. Click here for file

Additional file 4 **Comparison of results with and without cross-hybridization**. Fig. A.4 shows the importance ranking for thermodynamic properties with and without cross-hybridization. Click here for file

Additional file 5 **Stability of probe and target structures**. Table A.1 shows basic features that differ in the two datasets, including the stability of probe and target structures. Click here for file

Additional file 6 **Influence of probe-probe dimers**. Fig. A.5 shows the relative importance of Probe-Probe dimerization for the prediction of signal intensities. Click here for file
