# Supplementary material for: Hybridization thermodynamics of NimbleGen Microarrays
Source: BMC Bioinformatics. 2010 Jan 19;11:35. doi: 10.1186/1471-2105-11-35 (PMC2823707; doi:10.1186/1471-2105-11-35)
Supplement: Additional file 1 — Robustness of conclusions under scale transformation. Fig. A.1 shows the importance ranking for thermodynamic properties of probes without cross-hybridization against known targets on different intensity scales (linear and logarithmic). [file 1471-2105-11-35-S1.PDF]

A Additional File 1

Robustness of conclusions under scale transformations

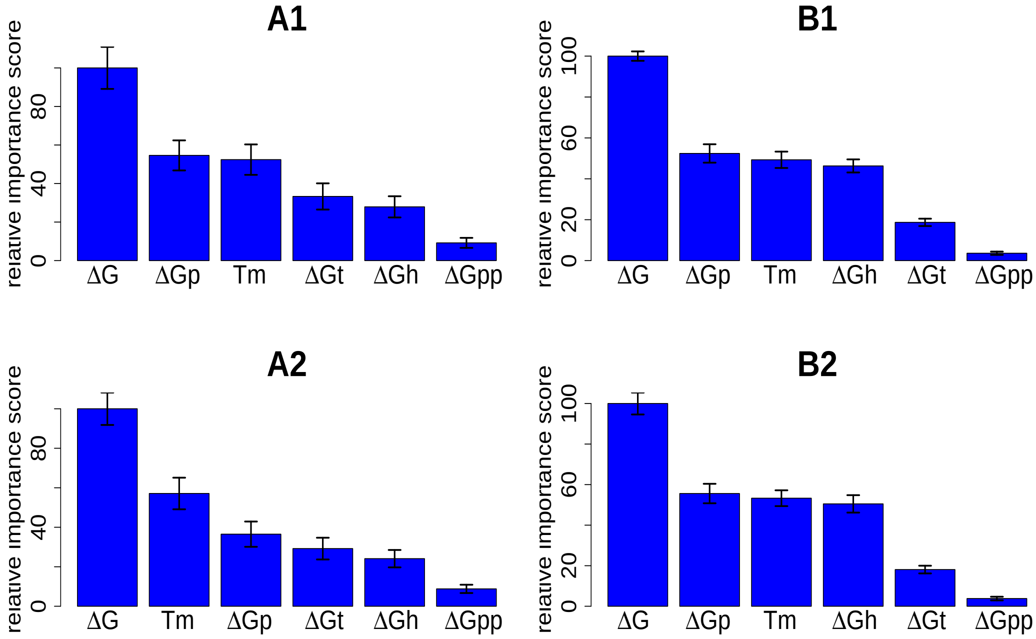

Figure A.1. The upper part of the figure shows the importance ranking for intensity prediction using a linear intensity scale (A1 and B1), in the lower part of the figure a logarithmic intensity is scale is used (A2 and B2). The left hand side (A1 and A2) shows the Wei et al. (1) data: Probes for clearly expressed transcripts without crosshybridization. The right hand side (B1 and B2) the dataset of Pozhitkov et al. (2).

To test the influence of different intensity scales on the GUIDE results, we used a linear intensity scale (see upper part of Fig. A.1, A1 and B1) and a logarithmic intensity scale (see lower part of Fig. A.1, A2 and B2) for GUIDE importance ranking. The influence of a linear versus a logarithmic intensity scale on the GUIDE results of the Wei et al. (1) dataset is shown in the left part of Fig. A.1, (A1 versus A2), the influence on the Pozhitkov et al. (2) data can be found on the right site of Fig. A.1, (B1 versus B2). Fig. A.1 shows that the effective interaction energy, ΔG, is the best predictor for signal intensity variation, independent of different intensity scales.

References

[1] Wei H, Kuan PF, Tian S, Yang C, Nie J, Sengupta S, Ruotti V, Jonsdottir GA, Keles S, Thomson JA, Stewart R: **A study of the relationships between oligonucleotide**

properties and hybridization signal intensities from NimbleGen microarray datasets. *Nucleic Acids Res* 2008, **36**:2926–38, [http://dx.doi.org/10.1093/nar/gkn133].

[2] Pozhitkov A, Noble PA, Domazet-Loso T, Nolte AW, Sonnenberg R, Staehler P, Beier M, Tautz D: **Tests of rRNA hybridization to microarrays suggest that hybridization characteristics of oligonucleotide probes for species discrimination cannot be predicted.** *Nucleic Acids Res* 2006, **34**(9):e66, [http://dx.doi.org/10.1093/nar/gkl133].
