# Supplementary material for: Hybridization thermodynamics of NimbleGen Microarrays
Source: BMC Bioinformatics. 2010 Jan 19;11:35. doi: 10.1186/1471-2105-11-35 (PMC2823707; doi:10.1186/1471-2105-11-35)
Supplement: Additional file 2 — Dominance of non-specific probes on the chip. Fig. A.2 shows the importance ranking of Tm, the mfe of probe structure, and probe length for all probes that had no target in a mature mRNA. [file 1471-2105-11-35-S2.PDF]

A Additional File 2

Dominance of non-specific Probes on the Chip

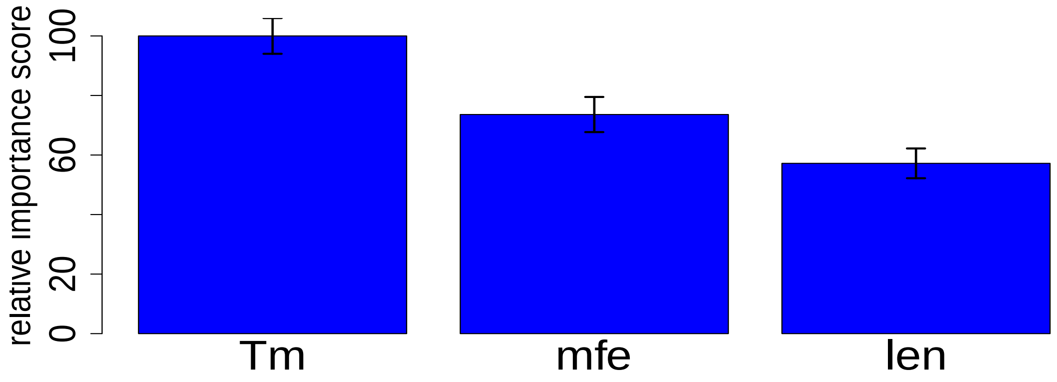

Figure A.2. Non-specific probes: Importance ranking of T_m, the minimal free energy (mfe) of probe structure, and probe length. Here, 'Tm' stands for the melting temperature, 'mfe' labels the minimal free energy of probe secondary structure and 'len' the probe length. Probe lengths ranged from 45 to 74 nt. Error bars are from 100 random bootstrap samples of 200,000 probes each.

Fig. A.2 shows the importance ranking of T_m, the mfe of probe structure, and probe length for all probes that hit a non-exonic region, *i. e.*, had no target in a mature mRNA. The agreement of Fig. 1 the main text and Fig. A.2 in the Supplement shows the dominance of non-specific effects on the chip. Consequently, the observations made reflect non-specific hybridization.
