# Supplementary material for: Hybridization thermodynamics of NimbleGen Microarrays
Source: BMC Bioinformatics. 2010 Jan 19;11:35. doi: 10.1186/1471-2105-11-35 (PMC2823707; doi:10.1186/1471-2105-11-35)
Supplement: Additional file 3 — Effect of intensity threshold. Fig. A.3 shows the importance ranking for thermodynamic properties of probes without cross-hybridization against known targets with different signal intensity thresholds. [file 1471-2105-11-35-S3.PDF]

A Additional File 3

Effect of Signal Intensity Threshold

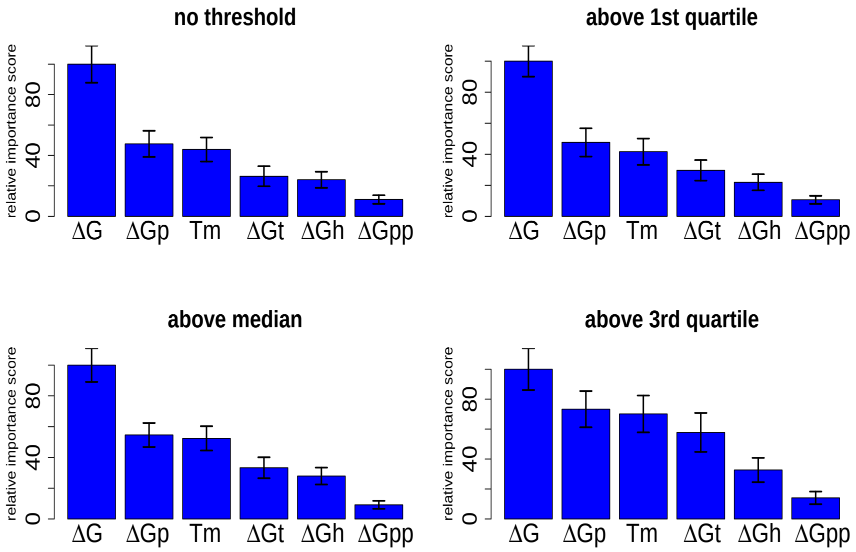

Figure A.3. Probes for known targets without crosshybridisation: Importance ranking for thermodynamic properties with different signal intensity thresholds.

In the Wei et al. (1) dataset the probes were hybridized with a cDNA solution of unknown composition. This cDNA solution represents various different targets, present in unknown concentrations. However, target expression level is an important factor for determining signal intensity. The influence of target concentration threshold on the importance ranking returned by GUIDE was tested using subsets with different thresholds for the minimal target concentration, see Fig A.3.

In the top left-hand corner of Fig A.3 we applied no threshold for target concentration. For the box plot in the top right corner of Fig A.3 the target concentration was above the 1st quartile of all targets. The targets in the bottom left plot had a concentration above the median and the ones in the bottom right plot had a concentration above the 3rd quartile of target concentration. In the subset above the 3rd quartile of target concentration the higher relative importance of the alternative thermodynamic parameters compared to ΔG is an effect of pronounced saturation of signal intensities for high target concentrations. Fig A.3 shows that a change in target concentration has no effect on the importance ranking of the different thermodynamic parameters.

References

[1] Wei H, Kuan PF, Tian S, Yang C, Nie J, Sengupta S, Ruotti V, Jonsdottir GA, Keles S, Thomson JA, Stewart R: **A study of the relationships between oligonucleotide properties and hybridization signal intensities from NimbleGen microarray datasets.** *Nucleic Acids Res* 2008, **36**:2926–38, [http://dx.doi.org/10.1093/nar/gkn133].
