# Supplementary material for: Hybridization thermodynamics of NimbleGen Microarrays
Source: BMC Bioinformatics. 2010 Jan 19;11:35. doi: 10.1186/1471-2105-11-35 (PMC2823707; doi:10.1186/1471-2105-11-35)
Supplement: Additional file 4 — Comparison of results with and without cross-hybridization. Fig. A.4 shows the importance ranking for thermodynamic properties with and without cross-hybridization. [file 1471-2105-11-35-S4.PDF]

A Additional File 4

Comparison of results with and without crosshybridization

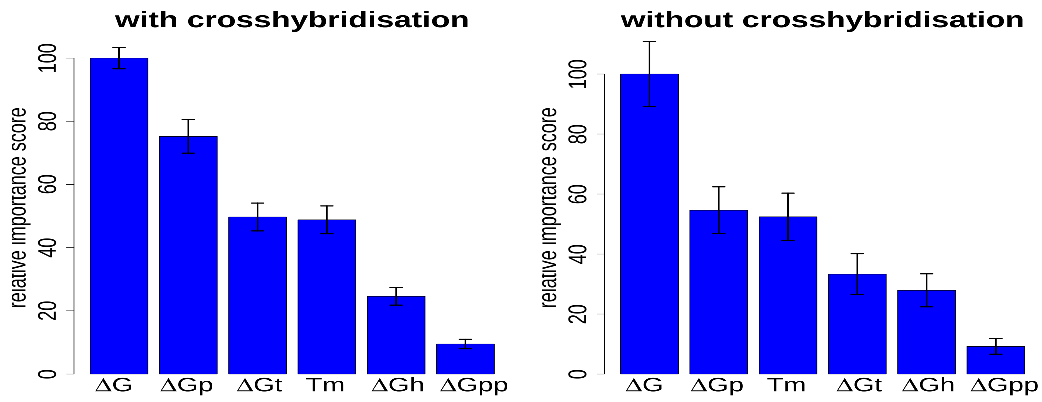

Figure A.4. Wei et al. dataset: Probes for clearly expressed transcripts: Importance ranking for thermodynamic properties with and without crosshybridization.

The Wei et al. (1) dataset comprises tiling probes that were not designed to avoid crosshybridization. Two thirds of the probes for clearly expressed genes show crosshybridization. Fig A.4 shows the results of GUIDE ranking for probes against clearly expressed transcripts, where probes with and without crosshybridisation are shown separately. The left hand side of Fig A.4 shows the results for probes with crosshybridization, the right hand side the results for probes that show no crosshybridization. We were interested to determine how crosshybridization influences the results of the GUIDE ranking: In both datasets the effective interaction energy ΔG is the best predictor for signal intensity variation. The ranking of ΔG, ΔG_h, and ΔG_{pp} is identical for probes with and without crosshybridization.

The relative importance of ΔG_p, the secondary structure of the probe, is significantly higher for probes with crosshybridization, where it is 65% compared to 55% for probes that show no crosshybridization. The higher relative importance of ΔG_p for probes with crosshybridization results from the fact, that probe secondary structure affects the intended perfectly complementary target and any off-target sequence in a comparable way: A highly stable secondary structure will interfere with the hybridisation of a perfectly complementary sequence as well as a sequence showing mismatches. This is not the case for other thermodynamic parameters. ΔG_h, the Tm, and ΔG_t are exclusively specific for the interaction of the probe with the perfectly complementary target sequence and give no information about the behaviour of off-target sequences. However, the signal of a probes showing crosshybridization is a combination of signals from different probe–target and probe–crossmatch pairs. Therefore,

thermodynamic parameters that are specifically characterising the probe–target interaction are expected to be less predictive than ΔG_p which interferes with the binding of target as well as off-target sequences.

References

[1] Wei H, Kuan PF, Tian S, Yang C, Nie J, Sengupta S, Ruotti V, Jonsdottir GA, Keles S, Thomson JA, Stewart R: **A study of the relationships between oligonucleotide properties and hybridization signal intensities from NimbleGen microarray datasets.** *Nucleic Acids Res* 2008, **36**:2926–38, [http://dx.doi.org/10.1093/nar/gkn133].
