# Supplementary material for: Hybridization thermodynamics of NimbleGen Microarrays
Source: BMC Bioinformatics. 2010 Jan 19;11:35. doi: 10.1186/1471-2105-11-35 (PMC2823707; doi:10.1186/1471-2105-11-35)
Supplement: Additional file 5 — Stability of probe and target structures. Table A.1 shows basic features that differ in the two datasets, including the stability of probe and target structures. [file 1471-2105-11-35-S5.PDF]

A Additional File 5

Comparison of Datasets

Since our model considers multiple inter- and intramolecular structures that compete with the formation of a probe–target duplex, the stability of structures within probes and targets is an important factor. The stability of a secondary structure is described by its free energy ΔG: The smaller ΔG (i.e., the more negative) the more stable is the structure. The median free energy bracketed by the free energy range of probe and target structures for both datasets is given in Table A.1. Table A.1 shows that stable structures interfering with probe–target binding were observed for most probe and target sequences. The free energies of ΔG_p, ΔG_t and ΔG_{pp} are always ≤ 0.

The dataset of Pozhitkov et al. (1) consists of probes targeting ribosomal RNAs (rRNAs). Ribosomal RNAs are known to form more stable structures than mRNAs. In addition the probes are considerably shorter than in the Wei et al. (2) dataset. Therefore 2% of the probe–target pairs have a $\Delta G \geq 0$, see Table A.1. Probe–target pairs with a $\Delta G \geq 0$ do not form a stable hybridization and were excluded from further analysis. In the dataset compiled from Wei et al. (2), 100% of all probe–target pairs had a negative ΔG, from the Pozhitkov et al. (1) dataset, 98% of the original set was retained.

References

[1] Pozhitkov A, Noble PA, Domazet-Loso T, Nolte AW, Sonnenberg R, Staehler P, Beier M, Tautz D: **Tests of rRNA hybridization to microarrays suggest that hybridization characteristics of oligonucleotide probes for species discrimination cannot be predicted.** *Nucleic Acids Res* 2006, **34**(9):e66, [http://dx.doi.org/10.1093/nar/gkl133].

[2] Wei H, Kuan PF, Tian S, Yang C, Nie J, Sengupta S, Ruotti V, Jonsdottir GA, Keles S, Thomson JA, Stewart R: **A study of the relationships between oligonucleotide properties and hybridization signal intensities from NimbleGen microarray datasets.** *Nucleic Acids Res* 2008, **36**:2926–38, [http://dx.doi.org/10.1093/nar/gkn133].

Comparison of datasets		
Parameter	Pozhitkov et al. (1)	Wei et al. (2)
probe	DNA	DNA
target	rRNA	cDNA
probe length	25-mers	45- to 75-mers
resolution	1 nt	22 nt
number of probes	7,519 [a]	21,813 [b]
number of targets	9 [a]	2,472 [b]
target concentration	375 ng	unknown
free energy ΔG in kcal/mol; [min., median, max.]		
ΔG	[− [c], −7.0, −23.3]	[−3.2, −45.6, −66.8]
ΔG of probe–target binding		
ΔG_h	[−0.7, −28.6, −43.5]	[−41.2, −66.2, −89.1]
ΔG of interfering structures		
ΔG_p	[− [d], −1.7, −9.2]	[−0.2, −6.3, −27.4]
ΔG_{pp}	[− [e], −1.5, −12.2]	[− [f], −2.6, −12.1]
ΔG_t	[− [g], −14.3, −33.5]	[−0.7, −10.7, −32.3]

[a] We analysed the probes perfectly matching the respective separately hybridized individual targets, ensuring no cross-hybridization effects.
[b] Our analysis focused on targeting clearly expressed transcripts and showing no cross-hybridization.
[c] For 2% of the probe–target pairs, the free energy of the sum of interfering structures was higher than or identical to the free energy of the probe–target duplex, giving $\Delta G \geq 0$, and these probes–target pairs where excluded from the analysis.
[d] 0.3% of probes had no stable secondary structure.
[e] 14% of probes formed no stable probe–probe dimers.
[f] 2% of probes formed no stable probe–probe dimers.
[g] 0.4% of targets had no stable secondary structure.

Table A.1
Summary of the characteristic differences between the two datasets used in this study. The dataset of Pozhitkov et al. (1) measures 1-nt tiling probes targeting rRNA fragments. The dataset of Wei et al. (2) consists of high-resolution tiling probes designed to interrogate the entire human genome. The row labelled ‘probes’ shows the nucleic acid type of the probes, the row labelled ‘target’ gives the nucleic acid type of the targets molecules. Each of the bottom rows shows the median free energy bracketed by the range of the free energy for the different thermodynamic parameters named in the first column. No values (−) are given for structures with non-negative free energies because they are are not stable.
