# Supplementary material for: Hybridization thermodynamics of NimbleGen Microarrays
Source: BMC Bioinformatics. 2010 Jan 19;11:35. doi: 10.1186/1471-2105-11-35 (PMC2823707; doi:10.1186/1471-2105-11-35)
Supplement: Additional file 6 — Influence of probe-probe dimers. Fig. A.5 shows the relative importance of Probe-Probe dimerization for the prediction of signal intensities. [file 1471-2105-11-35-S6.PDF]

A Additional File 6

Influence of ΔGpp

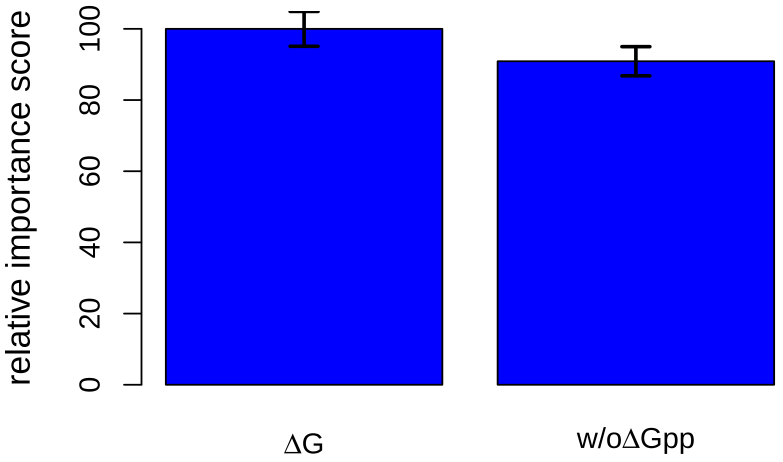

Figure A.5. Clearly expressed genes: The effect of ΔGpp for signal intensity prediction is determined by comparing the effective interaction free energy, ΔG, to the interaction energy without consideration of the free energy of probe–probe dimers, w/oΔGpp.

To determine the relative importance of ΔGpp for the prediction of signal intensities, we compared the effective interaction free energy, ΔG to the interaction free energy, that does not contain ΔGpp, i.e. $\Delta G_h - \Delta G_p - \Delta G_t$ (Suppl. Fig. A.5). When considering only the interference of the secondary structures of probe and target on probe–target duplex formation we come up to 90% of the predictive power of the effective interaction energy ΔG. Inclusion of the free energy of probe-probe dimerization, ΔG_{pp}, adds another 10% of predictive power to ΔG.
